# The Ideal Timing of Bilateral Total Knee Arthroplasty: Simultaneous Versus Staged

**DOI:** 10.22038/ABJS.2023.74559.3454

**Published:** 2024

**Authors:** Joseph.M Serino, E. Bailey Terhune, Robert A. Burnett, John D.D. Higgins, Joshua J. Jacobs, Craig J. Della Valle, Denis Nam

**Affiliations:** 1 Department of Orthopaedic Surgery, Rush University Medical Center, Chicago, IL, USA

**Keywords:** Complications, Knee arthritis, Outcomes, Timing, Total knee arthroplasty

## Abstract

**Objectives:**

The ideal timing for patients undergoing bilateral total knee arthroplasty (TKA) remains unknown. The purpose of this study was to compare 90-day outcomes between unilateral, simultaneous bilateral, and staged bilateral TKA.

**Methods:**

The PearlDiver database was used to retrospectively identify 231,119 patients undergoing primary TKA during 2015-2020, of which 67,956 (29.4%) were bilateral. Bilateral TKA patients were divided into cohorts of simultaneous bilateral TKA and staged bilateral TKA at 1-14 days, 15-30 days, 31-90 days, and 91-365 days. Each bilateral TKA cohort underwent one-to-one matching with unilateral TKA patients based on age, gender, year, Elixhauser Comorbidity Index (ECI), and a history of obesity, diabetes, and tobacco use. Ninety-day outcomes were compared between matched groups via univariate and multivariate analysis. In staged bilateral TKA groups, outcomes were collected beginning after the second TKA.

**Results:**

Compared to unilateral TKA, simultaneous bilateral TKA was associated with higher rates of venous thromboembolism (VTE; odds ratio [OR] 1.28, 95% confidence interval [CI] 1.07-1.54, p=0.007), acute kidney injury (AKI; OR 1.47, CI 1.17-1.84, p=0.001), blood transfusion (OR 6.81, CI 5.43-8.65, p<0.001), and any complication (OR 1.63, CI 1.49-1.78, p<0.001). Staged bilateral TKA at any time interval studied was associated with a similar or decreased risk of individual complications, emergency department visits, readmissions, reoperations, and any complication relative to unilateral TKA.

**Conclusion:**

Simultaneous bilateral TKA is associated with an increased risk of adverse events compared to unilateral TKA. However, bilateral TKA staged at a short interval appears safe in appropriately selected patients.

## Introduction

Bilateral knee osteoarthritis is seen in up to 5% of all adults over age 45 years and accounts for more than half of all patients with knee arthritis, especially among patients with known risk factors such as obesity or prior knee injuries.^1^^,^^ 2^ Approximately 43-93% of patients indicated for total knee arthroplasty (TKA) will require bilateral procedures within 5 years.^3^ Even in patients with unilateral disease, up to 21% will develop contralateral knee arthritis and 5% will require total knee arthroplasty (TKA) within 7 years.^4^

The optimal timing for bilateral TKA to ensure safe yet expedient care of patients remains controversial, particularly whether simultaneous or staged bilateral procedures are preferred. Prior studies have found that among all TKA procedures, 5-7% are simultaneous bilateral and 11-15% are staged bilateral within one year.^5^^-^^7^ Arguments in favor of simultaneous bilateral TKA include a single anesthetic, rehabilitation period, and hospitalization, resulting in decreased costs, better patient satisfaction, and quicker recovery.^8^^-^^11^ some studies have also found that simultaneous bilateral TKA is associated with lower rates of periprosthetic joint infection (PJI) and similar rates of other 

complications compared to staged bilateral procedures.^6^^, ^^12^^-^^17^ However, critics of simultaneous bilateral TKA point to significantly increased postoperative morbidity and mortality compared to staged bilateral TKA, particularly stroke, cardiac arrest, respiratory complications, confusion, thromboembolic events, and transfusion.^12^^, ^^18^^-^^23^

Currently, guidelines from the American Academy of Orthopaedic Surgeons (AAOS) and Consensus Conference on Bilateral TKA suggest that simultaneous bilateral TKA may be appropriate in select patients who are young and healthy.^24^^,^^ 25^ However, there is a paucity of evidence to guide the ideal time interval for staged procedures. Therefore, the purpose of this study was to compare 90-day outcomes between unilateral, simultaneous bilateral, and staged bilateral TKA to determine the optimal timing of bilateral TKA.

## Materials and Methods

The PearlDiver Mariner database (PearlDiver Inc., Fort Wayne, IN) was used to retrospectively identify patients undergoing primary TKA during 2015-2020. This database includes administrative claims data from over 144 million geographically diverse patients with Medicare, Medicaid, and private insurance. All data is completely de-identified, and therefore this study was considered exempt from our Institutional Review Board (IRB). Patients were identified using Current Procedural Terminology (CPT) code 27447 and laterality-specific International Classification of Disease, 10^th^ Revision (ICD-10) codes for primary TKA. To ensure equivalent follow-up for all patients and minimize attrition bias, only patients who were active within the database for a minimum of 6 months preoperatively until 3 months after their most recent primary TKA were included. 

Simultaneous bilateral procedures were identified by having an ICD-10 procedure code for a left TKA and right TKA on the same day. Staged procedures were identified as having these codes on different days. These patients were divided into cohorts of staged bilateral TKA at 1-14 days, 15-30 days, 31-90 days, and 91-365 days. Unilateral patients had a primary TKA code only once during the study period. Each cohort of bilateral TKA patients underwent 1:1 matching with unilateral TKA patients based on age, gender, year, Elixhauser Comorbidity Index (ECI), and a history of obesity, diabetes, and tobacco use. Patient characteristics and 90-day outcomes, including medical complications, mortality, reoperations, emergency department (ED) visits, and readmissions were compared between matched bilateral and unilateral TKA groups. “Any complication” was defined as any patient that experienced at least one adverse event. In staged bilateral TKA groups, outcomes were collected beginning after the second TKA. Although this method does not capture events occurring between staged procedures, it allows for a better assessment of the risk introduced by the second TKA compared to a unilateral procedure.

All statistical analyses were performed with R version 3.6.0 (R Foundation for Statistical Computing, Vienna, Austria) within PearlDiver’s proprietary software. Student’s T-test and Chi-Squared tests were used for univariate comparisons of continuous and categorical variables, respectively, between matched and unmatched groups. Multivariate logistic regression was also used to compare outcomes between matched groups with covariates of age, ECI, gender, and a preoperative history of obesity, diabetes, and tobacco use. The significance level was set at p<0.05.

## Results

Of the 231,119 patients identified, 163,163 (71%) underwent unilateral TKA, 7,454 (3%) underwent simultaneous bilateral TKA, and 60,502 (26%) underwent bilateral TKA staged within 1 year. Compared to unilateral TKA patients, simultaneous bilateral TKA patients were on average significantly younger (mean age 63.4 vs. 66.2 years, p<0.001), healthier (mean ECI 4.5 vs. 5.6, p<0.001), and had lower rates of tobacco use, diabetes, and obesity. Patients undergoing staged bilateral TKA were younger and healthier than unilateral TKA patients but were older and had more comorbidities compared to simultaneous bilateral TKA patients [Table 1]. After matching, there were no significant differences in preoperative characteristics or comorbidities between unilateral and bilateral TKA groups [Table 2]. 

**Table 1:** Patient demographics of unmatched cohorts

	**Unilateral**	**Simultaneous**	**Staged 1-14 days**	**Staged 15-30 days**	**Staged 31-90 days**	**Staged 91-365 days**	**p value**
N	163163	7454	3167	1186	11953	44196	-
Female	103193 (63.2%)	4038 (54.2%)	1889 (59.6%)	629 (53.0%)	6797 (56.9%)	28799 (65.1%)	<0.001
Age	66.2 ± 9.1	63.4 ± 8.2	65.1 ± 8.7	63.3 ± 8.6	63.6 ± 8.4	66.0 ± 8.5	<0.001
ECI	5.56 ± 3.38	4.48 ± 2.9	4.91 ± 3.22	4.48 ± 3.09	4.67 ± 3.08	5.18 ± 3.27	<0.001
**Comorbidities**							
Tobacco Use	30912 (18.9%)	979 (13.1%)	606 (19.1%)	255 (21.5%)	2420 (20.2%)	8606 (19.5%)	<0.001
Diabetes	42002 (25.7%)	1362 (18.3%)	799 (25.2%)	268 (22.6%)	2782 (23.3%)	11783 (26.7%)	<0.001
Obesity	42564 (26.1%)	1719 (23.1%)	1073 (33.9%)	411 (34.7%)	4544 (38.0%)	16288 (36.9%)	<0.001

ECI, Elixhauser Comorbidity Index; Values expressed as N (%) or mean ± standard deviation

**Table 2:** Patient demographics of matched cohorts

	**Simultaneous**			**Staged 1-14 days**			**Staged15-30 days**			**Staged 31-90 days**			**Staged 91-365 days**		
	**Unilateral**	**Bilateral**	**p**	**Unilateral**	**Bilateral**	**p**	**Unilateral**	**Bilateral**	**p**	**Unilateral**	**Bilateral**	**p**	**Unilateral**	**Bilateral**	**p**
**N**	**7368**	**7368**	**-**	**3112**	**3112**	**-**	**1163**	**1163**	**-**	**11675**	**11675**	**-**	**39085**	**39085**	**-**
**Female**	**4005 (54.4%)**	**4005 (54.4%)**	**1.000**	**1858 (59.7%)**	**1858 (59.7%)**	**1.000**	**617 (53.1%)**	**617 (53.1%)**	**1.000**	**6685 (57.3%)**	**6685 (57.3%)**	**1.000**	**25814 (66.0%)**	**25814 (66.0%)**	**1.000**
**Age**	**63.6 ± 8.1**	**63.5 ± 8.0**	**0.451**	**65.3 ± 8.6**	**65.3 ± 8.5**	**1.000**	**63.6 ± 8.3**	**63.5 ± 8.3**	**0.771**	**63.8 ± 8.3**	**63.8 ± 8.2**	**1.000**	**66.3 ± 8.2**	**66.3 ± 8.2**	**1.000**
**ECI**	**4.44 ± 2.83**	**4.44 ± 2.83**	**1.000**	**4.84 ± 3.11**	**4.84 ± 3.11**	**1.000**	**4.46 ± 3.04**	**4.46 ± 3.04**	**1.000**	**4.61 ± 2.97**	**4.61 ± 2.97**	**1.000**	**4.99 ± 3.01**	**4.99 ± 3.01**	**1.000**
**Comorbidities**															
**Tobacco Use**	**943 (12.8%)**	**943 (12.8%)**	**1.000**	**576 (18.5%)**	**576 (18.5%)**	**1.000**	**240 (20.6%)**	**240 (20.6%)**	**1.000**	**2267 (19.4%)**	**2267 (19.4%)**	**1.000**	**6770 (17.3%)**	**6770 (17.3%)**	**1.000**
**Diabetes**	**1333 (18.1%)**	**1333 (18.1%)**	**1.000**	**773 (24.8%)**	**773 (24.8%)**	**1.000**	**257 (22.1%)**	**257 (22.1%)**	**1.000**	**2665 (22.8%)**	**2665 (22.8%)**	**1.000**	**9728 (24.9%)**	**9728 (24.9%)**	**1.000**
**Obesity**	**1673 (22.7%)**	**1673 (22.7%)**	**1.000**	**1034 (33.2%)**	**1034 (33.2%)**	**1.000**	**398 (34.2%)**	**398 (34.2%)**	**1.000**	**4351 (37.3%)**	**4351 (37.3%)**	**1.000**	**13438 (34.4%)**	**13438 (34.4%)**	**1.000**

ECI, Elixhauser Comorbidity Index; Values expressed as N (%) or mean ± standard deviation

Simultaneous bilateral TKA was associated with significantly higher rates of postoperative venous thromboembolism (VTE; 3.7% vs. 2.9%, p=0.010), acute kidney injury (AKI; 2.6% vs. 1.8%, p=0.001), blood transfusion (7.2% vs. 1.2%, p<0.001), urinary tract infection (UTI; 4.0% vs. 3.1%, p=0.004), and any complication (21.0% vs. 14.2%, p<0.001) compared to unilateral TKA [Table 3]. After multivariate analysis, simultaneous bilateral TKA was independently associated with an increased risk of VTE (odds ratio [OR] 1.28, 95% confidence interval [CI] 1.07-1.54, p=0.007), acute kidney injury (AKI; OR 1.47, CI 1.17-1.84, p=0.001), transfusion (OR 6.81, CI 5.43-8.65, p<0.001), UTI (OR 1.31, CI 1.10-1.57, p=0.002) and any complication (OR 1.63, CI 1.49-1.78, p<0.001). Patients undergoing simultaneous bilateral TKA experienced similar rates of PJI, reoperations, ED visits, and readmissions relative to unilateral TKA [Table 4].

Bilateral TKA staged at 1-14 days was associated with similar 90-day rates of all adverse events compared to unilateral TKA. When staged at 15-30 days, bilateral TKA resulted in similar rates of individual complications and a significantly lower risk of any complication (OR 0.69, CI 0.38-1.22, p=0.045) relative to unilateral TKA. Staged bilateral TKA at 31-90 days and 91-365 days was also found to have a similar or decreased risk of individual complications, ED visits, readmissions, reoperations, and any complication compared to unilateral TKA.

**Table 3:** Univariate analysis of 90-day complications after unilateral and staged bilateral TKA

	**Simultaneous**			**Staged 1-14 days**			**Staged 15-30 days**			**Staged 31-90 days**			**Staged 91-365 days**		
	**Unilateral**	**Bilateral**	**p**	**Unilateral**	**Bilateral**	**p**	**Unilateral**	**Bilateral**	**p**	**Unilateral**	**Bilateral**	**p**	**Unilateral**	**Bilateral**	**p**
**Superficial Site Infection**	**85 ** **(1.2%)**	**79** ** (1.1%)**	**0.695**	**46 ** **(1.5%)**	**36 ** **(1.2%)**	**0.317**	**11 ** **(0.9%)**	**13 (1.1%)**	**0.837**	**142 (1.2%)**	**79 ** **(0.7%)**	**<0.001**	**542** ** (1.4%)**	**414 ** **(1.1%)**	**<0.001**
**PJI**	**66** ** (0.9%)**	**51** **(0.7%)**	**0.194**	**30 ** **(1.0%)**	**35 ** **(1.1%)**	**0.618**	**6 ** **(0.5%)**	**11 (0.9%)**	**0.330**	**100 (0.9%)**	**88 ** **(0.8%)**	**0.421**	**357** ** (0.9%)**	**334** ** (0.9%)**	**0.401**
**VTE**	**214 ** **(2.9%)**	**271 (3.7%)**	**0.010**	**87** ** (2.8%)**	**98 ** **(3.1%)**	**0.455**	**35 ** **(3.0%)**	**21 (1.8%)**	**0.079**	**331 (2.8%)**	**179** ** (1.5%)**	**<0.001**	**1228 (3.1%)**	**898** ** (2.3%)**	**<0.001**
**Acute Kidney Injury**	**134** ** (1.8%)**	**192 (2.6%)**	**0.001**	**59** ** (1.9%)**	**63 ** **(2.0%)**	**0.784**	**22 ** **(1.9%)**	**14 (1.2%)**	**0.240**	**240 (2.1%)**	**126** ** (1.1%)**	**<0.001**	**867 ** **(2.2%)**	**681** ** (1.7%)**	**<0.001**
**Cardiac Arrest**	**3** ** (0.0%)**	**8 ** **(0.1%)**	**0.228**	**0 ** **(0.0%)**	**1** ** (0.0%)**	**1.000**	**0 ** **(0.0%)**	**0 (0.0%)**	**1.000**	**7 ** **(0.1%)**	**1** ** (0.0%)**	**0.045**	**13** ** (0.0%)**	**16 ** **(0.0%)**	**0.710**
**Wound Dehiscence**	**80** ** (1.1%)**	**70 ** **(1.0%)**	**0.460**	**35 ** **(1.1%)**	**48** ** (1.5%)**	**0.185**	**10** ** (0.9%)**	**11 (0.9%)**	**1.000**	**124 (1.1%)**	**108** ** (0.9%)**	**0.322**	**453 ** **(1.2%)**	**434** ** (1.1%)**	**0.543**
**Hematoma**	**24 ** **(0.3%)**	**34 ** **(0.5%)**	**0.236**	**21 ** **(0.7%)**	**14 ** **(0.4%)**	**0.309**	**6** ** (0.5%)**	**2 (0.2%)**	**0.288**	**55 (0.5%)**	**27** ** (0.2%)**	**0.003**	**173** ** (0.4%)**	**143 ** **(0.4%)**	**0.102**
**Pneumonia**	**73 ** **(1.0%)**	**85** ** (1.2%)**	**0.379**	**41** ** (1.3%)**	**37** ** (1.2%)**	**0.733**	**10** ** (0.9%)**	**11 (0.9%)**	**1.000**	**102 (0.9%)**	**66 ** **(0.6%)**	**0.007**	**437 ** **(1.1%)**	**336** ** (0.9%)**	**<0.001**
**Transfusion**	**85** ** (1.2%)**	**529 (7.2%)**	**<0.001**	**55 ** **(1.8%)**	**51** ** (1.6%)**	**0.769**	**12 ** **(1.0%)**	**7 (0.6%)**	**0.357**	**143 (1.2%)**	**55** ** (0.5%)**	**<0.001**	**495 ** **(1.3%)**	**290** ** (0.7%)**	**<0.001**
**Urinary Tract Infection**	**229** ** (3.1%)**	**295 (4.0%)**	**0.004**	**116 (3.7%)**	**109 (3.5%)**	**0.684**	**26** ** (2.2%)**	**18 (1.5%)**	**0.287**	**335 (2.9%)**	**257 ** **(2.2%)**	**0.001**	**1482 (3.8%)**	**1195 (3.1%)**	**<0.001**
**Mortality**	**0 ** **(0.0%)**	**0 ** **(0.0%)**	**1.000**	**0 (0.0%)**	**3** ** (0.1%)**	**0.248**	**0 ** **(0.0%)**	**0 (0.0%)**	**1.000**	**0** ** (0.0%)**	**2 ** **(0.0%)**	**0.480**	**3** ** (0.0%)**	**0 ** **(0.0%)**	**1.000**
**Reoperation**	**69 ** **(0.9%)**	**56** ** (0.8%)**	**0.281**	**36 (1.2%)**	**39** ** (1.3%)**	**0.816**	**8 ** **(0.7%)**	**7 (0.6%)**	**1.000**	**104 (0.9%)**	**116 ** **(1.0%)**	**0.456**	**368** ** (0.9%)**	**385 ** **(1.0%)**	**0.558**
**ED Visit**	**296** ** (4.0%)**	**287 (3.9%)**	**0.735**	**125 (4.0%)**	**102 (3.3%)**	**0.137**	**44 ** **(3.8%)**	**29 (2.5%)**	**0.096**	**465 (4.0%)**	**242** ** (2.1%)**	**<0.001**	**1568 (4.0%)**	**1016 (2.6%)**	**<0.001**
**Readmission**	**163** ** (2.2%)**	**175 (2.4%)**	**0.545**	**81 ** **(2.6%)**	**81 ** **(2.6%)**	**1.000**	**29** ** (2.5%)**	**20 (1.7%)**	**0.248**	**267 (2.3%)**	**153** ** (1.3%)**	**<0.001**	**972 ** **(2.5%)**	**483** ** (1.2%)**	**<0.001**
**Any Complication**	**1048 (14.2%)**	**1550 (21.0%)**	**<0.001**	**477 (15.3%)**	**486 (15.6%)**	**0.779**	**155 (13.3%)**	**123 (10.6%)**	**0.048**	**1644 (14.1%)**	**1077 (9.2%)**	**<0.001**	**5998 (15.3%)**	**4139 (10.6%)**	**<0.001**

PJI; periprosthetic joint infection; VTE, venous thromboembolism; ED, Emergency Department

**Table 4:** Multivariate analysis of 90-day complications after unilateral and staged bilateral TKA. Odds ratios are expressed relative to unilateral TKA

	**Simultaneous**		**Staged 1-14 days**		**Staged 15-30 days**		**Staged 31-90 days**		**Staged 91-365 days**	
	**OR (95 CI %)**	**p**	**OR (95 CI %)**	**p**	**OR (95 CI %)**	**p**	**OR (95 CI %)**	**p**	**OR (95 CI %)**	**p**
Superficial Site Infection	0.93 (0.68 - 1.27)	0.649	0.78 (0.50 - 1.21)	0.275	1.19 (0.53 - 2.72)	0.675	0.56 (0.42 - 0.73)	<0.001	0.69 (0.61 - 0.79)	<0.001
PJI	0.77 (0.53 - 1.11)	0.167	1.17 (0.72 - 1.93)	0.525	1.85 (0.70 - 5.38)	0.229	0.88 (0.66 - 1.18)	0.393	0.89 (0.77 - 1.04)	0.153
VTE	1.28 (1.07 - 1.54)	0.007	1.14 (0.85 - 1.53)	0.393	0.59 (0.34 - 1.02)	0.064	0.54 (0.44 - 0.64)	<0.001	0.68 (0.62 - 0.74)	<0.001
Acute Kidney Injury	1.47 (1.17 - 1.84)	0.001	1.08 (0.75 - 1.56)	0.674	0.63 (0.31 - 1.24)	0.191	0.52 (0.42 - 0.65)	<0.001	0.74 (0.67 - 0.82)	<0.001
Cardiac Arrest	2.69 (0.78 - 12.28)	0.144	NA	-	NA	-	0.13 (0.02 - 1.01)	0.051	1.16 (0.54 - 2.49)	0.289
Wound Dehiscence	0.88 (0.63 - 1.21)	0.421	1.38 (0.90 - 2.16)	0.147	1.10 (0.46 - 2.66)	0.821	0.87 (0.67 - 1.13)	0.307	0.91 (0.79 - 1.04)	0.154
Hematoma	1.42 (0.85 - 2.43)	0.189	0.67 (0.33 - 1.30)	0.242	0.33 (0.05 - 1.45)	0.178	0.49 (0.31 - 0.77)	0.003	0.77 (0.61 - 0.97)	0.026
Pneumonia	1.17 (0.86 - 1.61)	0.317	0.91 (0.58 - 1.42)	0.670	1.12 (0.47 - 2.70)	0.803	0.65 (0.47 - 0.88)	0.007	0.74 (0.63 - 0.85)	<0.001
Transfusion	6.81 (5.43 - 8.65)	<0.001	0.93 (0.63 - 1.37)	0.714	0.58 (0.22 - 1.46)	0.262	0.38 (0.28 - 0.52)	<0.001	0.53 (0.46 - 0.62)	<0.001
Urinary Tract Infection	1.31 (1.10 - 1.57)	0.002	0.94 (0.72 - 1.23)	0.661	0.69 (0.37 - 1.27)	0.239	0.76 (0.65 - 0.90)	0.001	0.76 (0.70 - 0.83)	<0.001
Mortality	NA	-	NA	-	NA	-	NA	-	NA	-
Reoperation	0.81 (0.57 - 1.15)	0.246	1.09 (0.69 - 1.72)	0.719	0.88 (0.31 - 2.45)	0.797	1.11 (0.85 - 1.45)	0.448	1.01 (0.88 - 1.17)	0.849
ED Visit	0.97 (0.82 - 1.14)	0.704	0.81 (0.62 - 1.05)	0.115	0.65 (0.40 - 1.04)	0.075	1.14 (0.87 - 1.48)	0.337	0.59 (0.54 - 0.64)	<0.001
Readmission	1.08 (0.87 - 1.34)	0.487	1.01 (0.73 - 1.38)	0.975	0.69 (0.38 - 1.22)	0.203	0.57 (0.46 - 0.69)	<0.001	0.49 (0.43 - 0.54)	<0.001
Any Complication	1.63 (1.49 - 1.78)	<0.001	1.03 (0.89 - 1.18)	0.703	0.69 (0.38 - 1.22)	0.045	0.62 (0.57 - 0.67)	<0.001	0.64 (0.61 - 0.67)	<0.001

OR, odds ratio; CI, confidence interval; PJI; periprosthetic joint infection; VTE, venous thromboembolism; ED, Emergency Department; NA, not applicable, used where odds ratio could not be calculated due to 0 events in at least one group.

## Discussion

 We found that simultaneous bilateral TKA is associated with an increased risk of adverse events compared to unilateral TKA, including both medical and surgical complications. In particular, patients undergoing simultaneous bilateral TKA had a transfusion rate more than six times the matched unilateral TKA group. However, bilateral TKA staged as early as 1-14 days appears safe in appropriately selected patients, with no increased risk of individual or any complications relative to unilateral TKA. Therefore, our data suggests that bilateral TKAs may be safelyz staged at a short interval, or one that is most convenient to the surgeon and patient, whereas simultaneous bilateral procedures should be avoided whenever possible. 

 Prior studies have similarly associated simultaneous bilateral TKA with an increased risk of complications compared to both staged bilateral and unilateral TKA.^8^^-^^12^^, ^^18^^-^^23^ In a meta-analysis of 18 randomized controlled trials including 27,807 patients, Restrepo et al. found that simultaneous bilateral TKA resulted in increased rates of pulmonary embolism (OR 1.8), mortality (OR 2.2), and cardiac complications (OR 2.5) compared to unilateral TKA.^22^ However, there were no significant differences in outcomes after unilateral TKA and bilateral TKA staged at a minimum of three months. Simultaneous bilateral TKA has also been found to increase the risk of confusion, respiratory complications, transfusion, and discharge to nursing or rehabilitation facilities relative to unilateral or staged bilateral procedures.^9^^, ^^12^^, ^^18^^, ^^20^ Recently, Follet et al. reported that alignment outliers (defined as > 3˚ from the mechanical axis) were more than twice as common among the second TKA in simultaneous bilateral TKA compared to staged procedures.^26^

 The AAOS 2015 Clinical Practice Guidelines suggests based on limited evidence that simultaneous bilateral TKA can be safely performed in patients who are younger than 70 years old or have an American Society of Anesthesiologists (ASA) score of 1-2.^24^ However, the guidelines do not provide any guidance for the ideal timing of staged procedures in patients who do not meet their criteria. Similarly, the statement from the Consensus Conference on Bilateral TKA acknowledges the increased risk of morbidity and mortality with simultaneous bilateral TKA and supports its use only in patients meeting strict criteria, including age < 75 years, a revised cardiac risk index < 4,^27^ and no major comorbidities.^25^ The consensus statement suggests that for all other patients, bilateral procedures should be staged at a minimum of three months based only on theoretical grounds due to insufficient evidence.^25^

 However, our data suggests that bilateral TKA can be safely performed when staged as early as 1-14 days without increasing the risk of complications relative to a unilateral procedure. Previous studies have also supported staging procedures at a short time interval. Courtney et al. evaluated 131 patients undergoing bilateral TKA staged within 1 week and found no difference in complications, readmission, and reoperations at up to one year when compared to unilateral TKA.^28^ The authors argue that this short time interval provides the benefit of a single rehabilitation period without the increased medical risk of a simultaneous bilateral procedure, while also allowing the patient and surgeon to delay the second procedure if any unexpected complications arise.^28^ Sliva et al. similarly reported that bilateral TKA staged at 4-7 days during a single admission resulted in significantly fewer complications compared to simultaneous bilateral TKA.^29^ However, Medicare bundled-payment models still incentivize waiting a minimum of 91 days between procedures. Harrer et al. found that under the Bundled Payment for Care Improvement (BPCI) program, bilateral total joint arthroplasty staged within 90 days significantly increased episode-of-care costs by $2,021 and decreased 

the bundle savings margin by $2,868 per patient compared to procedures staged at 91-120 days, despite similar complication rates and discharge disposition.^30^ Such bundled-payment programs should rather align financial incentives with the current evidence that supports the safe and expeditious care of patients. Instead, patients may face unnecessary delays in their second procedure. 

 The present study has certain limitations. First, assessment of patient characteristics and adverse events is reliant on accurate coding within the PearlDiver database. Administrative claims data has been found to be highly specific though variably sensitive, suggesting that some complications may not be fully represented in the database.^31^ Second, functional outcomes are not available within the database, representing an important area for further research when determining the optimal timing of bilateral TKA. Third, because complications were collected beginning after the second TKA in staged procedures, we were more likely to capture complications associated with the first procedure in the staged 1-14 day group compared to the 91-365 day group. This may have overestimated the true complication rate associated with the second TKA. Our results should therefore be considered a conservative comparison between staged bilateral and unilateral TKA, which further supports the safety of staged bilateral procedures. Importantly, our study is subject to an inherent selection bias. Although matched groups and multivariate analysis were used to minimize the effect of potential confounders, there may be differences between groups not adequately captured by the matching process. Similarly, our study was not able to evaluate which patients are ideal candidates for simultaneous bilateral TKA or whether different time intervals for staged procedures are more appropriate for certain patients.

## Conclusion

In conclusion, despite an increased risk of complications with simultaneous bilateral TKA, staged procedures at an interval as short as 1-14 days appears safe in appropriately selected patients, with no increased risk of individual or any complications relative to unilateral TKA. Future studies should evaluate the selection criteria for these patients and determine if a patient-specific time interval exists. Bundled-payment reform may also be necessary to align incentives and ensure safe, expeditious care of patients with bilateral knee arthritis. 
